# The impact of Medicare prescription drug coverage on the use of antidementia drugs

**DOI:** 10.1186/1471-2318-13-37

**Published:** 2013-04-27

**Authors:** Nicole R Fowler, Yi-Fan Chen, Christiana A Thurton, Aiju Men, Eric G Rodriguez, Julie M Donohue

**Affiliations:** 1 Department of Medicine, School of Medicine, University of Pittsburgh, Pittsburgh, PA, USA; 2 Department of Biostatistics, Graduate School of Public Health, University of Pittsburgh, Pittsburgh, PA, USA; 3 Department of Community Health, CUNY School of Public Health at Hunter College, New York, NY, USA; 4 Department of Health Policy and Management, Graduate School of Public Health University of Pittsburgh, Pittsburgh, PA, USA

**Keywords:** Dementia, Alzheimer’s disease, Medicare Part D, Cholinesterase inhibitors, Memantine, Drug coverage

## Abstract

**Background:**

Cholinesterase inhibitors and memantine are prescribed to slow the progression dementia. Although the efficacy of these drugs has been demonstrated, their effectiveness, from the perspective of patients and caregivers, has been questioned. Little is known about whether the demand for cholinesterase inhibitors and memantine are sensitive to out-of-pocket cost. Using the 2006 implementation of Medicare Part D as a natural experiment, this study examines the impact of changes in drug coverage on use of cholinesterase inhibitors and memantine by comparing use before and after Medicare Part D implementation among older adults who did and did not experience a change in coverage.

**Methods:**

Retrospective analyses of claims data from 35,102 community-dwelling Medicare beneficiaries in Pennsylvania aged 65 or older. Beneficiaries were continuously enrolled in a Medicare Advantage plan from 2004 to 2007. Outcome variables were any use of donepezil (Aricept®), galantamine (Razadyne®), rivastigmine (Exelon®), tacrine (Cognex®), or memantine (Namenda®) each year and the number of 30-day prescriptions filled for these drugs. Independent variables included type of drug benefit pre–Part D (No coverage, $150 cap, $350 cap, and No cap as the reference group), time period, and their interaction. Sensitivity analyses were conducted to test if there are differences in use by drug class or if beneficiaries with a diagnosis of dementia pre–Part D experienced an increase in use post–Part D.

**Results:**

The No coverage group had a 38% increase in the odds ratio of any use of antidementia medications (*P* = 0.0008) post–Part D relative to the No cap group. All four coverage groups had significant increases in number of 30-day prescriptions (*P* < 0.001) over the study period. In adjusted models that included the sub-sample with any use pre–Part D, the No coverage group had a 36% increase in prescriptions (*P* = 0.002) and the $350 cap group had a 15% increase (*P* = 0.003) after adjusting for trends in the No cap group. Results from the sensitivity analysis for the sub-sample with a diagnosis of dementia pre–Part D show that each group had significant increases in 30-day prescriptions compared to the No cap control group (*P* < 0.05).

**Conclusions:**

Use of cholinesterase inhibitors and memantine in our sample increased and a greater increase in use was observed among Medicare beneficiaries who experienced improvements in drug coverage under Medicare Part D.

## Background

Dementia is a prevalent condition, affecting an estimated 5.4 million people, including 1 of every 8 adults 65 years or older [1]. The estimated societal costs of dementia include health care spending and lost productivity of caregivers is approximately US$200 billion [2]. Two classes of medication – cholinesterase inhibitors (donepezil, galantamine, rivastigmine, and tacrine) and a neuropeptide-modifying agent (memantine) – introduced to treat dementia have reached sales in excess of $1 billion. In 2008, it was estimated that 24.7% of older adults with dementia living in the community were taking a cholinesterase inhibitor or memantine (hereafter referred to antidementia drugs) [3,4], at an average out-of-pocket cost of US $166 [5].

There is considerable controversy over the value of antidementia medications as they are currently used. These agents slow the progression of cognitive decline but do not reverse the effects of the disease [6,7]. Randomized controlled trials of cholinesterase inhibitors have shown short–term positive effects on cognitive performance in patients with mild Alzheimer’s disease [8]. There is little evidence to support their use in patients with advanced disease [3,9], yet studies show that as much as 10-30% of use of these medications is among people with severe dementia living in nursing homes [10,11]. Furthermore, these drugs are primarily recommended for Alzheimer’s-type dementia, where the strongest evidence of efficacy has been found [7]. Finally, widespread use of these drugs has been questioned due a lack of evidence that they improve outcomes that are meaningful to patients and caregivers such as the rate of decline in activities of daily living and behavioral symptoms [7,12,13]. Perhaps as a result of this mixed evidence, prescribing guidelines for these medications are inconsistent [14-17], even for patients with mild cognitive impairment who may stand to benefit more than patients with severe impairment [18,19].

Population based rates of use for antidementia drugs vary, as do estimates of persistence of therapy overtime. Some studies have found that as many as 50% of people who initiate use of an antidementia medication discontinue use within 1 year [20]. Little is known about the effect of out-of-pocket costs on use of antidementia medications. This gap in knowledge is important given the significant expansion in drug coverage brought about by Medicare Part D, which was implemented in 2006 and covers drugs for 27 million beneficiaries. Previous studies have found Part D to increase both appropriate and inappropriate use of medications [21-24].

The objective of this study was to determine the effect of insurance coverage on the use of antidementia medications. This study had two hypotheses: first, that the overall proportion of patients using these drugs would increase over the study period; second, that the increase in use of these drugs would be higher among patients gaining prescription drug coverage under Part D.

## Methods

### Study sample

With approval from the University of Pittsburgh Institutional Review Board, we obtained and examined the pharmacy, inpatient and outpatient medical claims, and enrollment records of patients covered by a large health insurer in Pennsylvania from 2004–2007. The criteria for inclusion in the retrospective cohort study were community-dwelling Medicare beneficiaries who were 65 years or older and were alive and continuously enrolled from 2004 to 2007 in one of the Medicare managed care plans (Medicare Advantage) offered by the health insurer. The sample was limited to individuals who filled at least one prescription for any drug in one of several thousand pharmacies in the insurer’s network to ensure inclusion of fills even for those with no drug benefits pre–Part D. All enrollees in the plan, regardless of the level of coverage, had an incentive to present their insurance card at these pharmacies because they received a 15% discount. We are therefore confident that we observed prescription fills for these enrollees.

The strength of the data, compared to national Medicare data, is the availability of pre–Part D drug utilization and drug benefits as a way to compare use pre and post–Part D. Patients were categorized into 4 groups based on their pre–Part D drug coverage: those who had no drug coverage (No coverage group); those who had quarterly drug benefit limits on what the plan would pay of either $150 ($150 cap group) or $350 ($350 cap group), based solely on their county of residence; and those who were enrolled in employer or union group plans through the same insurer that offered supplemental drug coverage with no quarterly cap (No cap group). The $150 cap and $350 cap groups had copayments of $12 and $20 for generic and brand name drugs, respectively. The No cap group had copayments of $10 and $20, respectively. Other medical benefits, such as outpatient visit copayments, were similar for the 4 groups.

The implementation of Medicare Part D in January 2006 was used a natural experiment to examine the impact of changes in drug coverage on use of antidementia drugs. Use of these drugs was compared before and after this date in both the full study sample and among a sub-sample who had any use of an antidementia drug pre–Part D. When Part D went into effect, beneficiaries in the No coverage group and those in the $150 cap and $350 cap groups automatically received the plan’s Part D benefit. The coverage, like that of most Medicare Advantage prescription drug plans, did not include a deductible.

Beneficiaries had copayments on drugs until their total drug spending for the year reached the “donut hole” (the coverage gap for out-of-pocket payments from $2,250 to $5,100 in 2006 dollars). In the donut hole, the plan either covered no drugs or covered only generic drugs with an $8 or $10 copayment. In 2006, no antidementia drugs were generic, so beneficiaries with Part D coverage would have had to pay 100% of the costs of these drugs once they reached the donut hole. Beneficiaries in the No cap group had stable drug benefits throughout the study period and were not exposed to coverage limits and served as the comparison group. This comparison group accounts for secular trends in use of these drugs among beneficiaries enrolled with the same insurance company who did not experience a change in their drug benefit as a result of Medicare Part D.

### Outcomes measures, variables, and covariates

This study had two outcome measures: a population-based measure of any use of the 5 antidementia drugs approved by the Food and Drug Administration for the treatment of dementia: donepezil (Aricept®), galantamine (Razadyne®), rivastigmine (Exelon®), tacrine (Cognex®), or memantine (Namenda®); and a measure of the annual number of 30-day prescriptions filled for any of these medications conditional on any use pre–Part D.

Our primary independent variables were the type of drug benefit pre–Part D (No coverage, $150 cap, $350 cap, and No cap as the reference group), time period, and their interaction. Our covariates included the following: sex; age; census-block group-level data on race, proportion with incomes below the poverty line, urban residence; and three measures of health status. First, we included indicators for certain chronic conditions (binary variables for congestive heart failure, depression, diabetes, hypertension, and ischemic heart disease). Second, we included a time–varying prospective risk score, a proxy for health status. The risk scores were calculated at the end of each year with Risk Grouper (DxCG, Boston, MA), a software program that uses a series of proprietary algorithms based on the presence of dozens of ICD-9 diagnostic codes and Healthcare Common Procedure Coding System (HCPCS) codes. The scores are similar to the hierarchical condition category weights that are used by the Centers for Medicare and Medicaid Services to adjust Medicare Advantage Part D payments. A higher score indicates the likelihood of greater spending in the following year. Third, we included measures of the use of medical services (number of outpatient physician visits each year and outpatient costs).

### Statistical analyses

Descriptive statistics were used to characterize the study sample. To compare the characteristics of the 4 study groups, chi-square tests were used for categorical variables and *t* tests and multivariate analysis of variance (MANOVA) for continuous variables. To test the difference of two dependent proportions, McNemar’s test was used.

A generalized estimating equation (GEE) [25] with a binomial distribution, logit link function, and exchangeable correlation structure was fitted to model any use of antidementia drugs annually, as a group and by drug class. Similar models were fitted for the number of prescriptions annually, except using negative binominal distribution, and log link function. The negative binominal distribution dealt with the issue of over dispersion of the count, and the correlation structure took into account the dependency within patients. By putting the interaction terms of time period and the drug benefit group in the model, we were able to explore the net effects of the implementation of Part D within each coverage group adjusting for the covariates. This is achieved by comparing the adjusted odds ratio of the No coverage group, $150 cap group, and $350 cap group with that of the reference group (No cap group) to adjust for secular trends in the use of antidementia medications unrelated to Part D’s implementation.

Additionally, a sensitivity analysis was conducted using only the sub-sample of beneficiaries who had an ICD-9 diagnosis code for any type of dementia (e.g., Alzheimer’s disease, vascular dementia, senility, etc.) pre–Part D and compared use of antidementia drugs pre and post–Part D, by coverage group.

SAS version 9.2 (SAS Institute, Inc., Cary, NC) was used all analyses with the supplement of Excel 2007 for plots. Any *P* value of <0.05 was considered to be statistically significant.

## Results

The total study sample consisted of 35,102 patients. Of these 11.2% (n = 3,939) were in the No coverage group, 7.5% (n = 2,662) in the $150 cap group, 54.2% (n = 19,014) in the $350 cap group, and 27.0% (n = 9,487) in the No cap group (Table 1). In each group, there were more beneficiaries who were women (≥52.5%), aged 65–74 years (≥47.4%), and white (≥0.92), and with a median income above $34,000. Compared to the No cap group, the other three groups had a higher proportion of females (*P* ≤ 0.002), were more likely to be older than 75 years of age (*P* ≤ 0.001), and were more likely to be >200% below the poverty line (*P* ≤ 0.001), which reflects that these groups were less likely to have coverage through a former employer. The No coverage group had lower percentages of patients with comorbidities and lower outpatient visits and costs. However, these four groups had similar risk scores over time indicating comparable health status. None of the beneficiaries, in any group, had a claim for tacrine (Cognex®) during the study period. This is not surprising given its known side effects of high toxicity of enzymes in the liver [26]. Only beneficiaries in the $350 cap group and No cap group had claims for galantamine (Razadyne®) in the pre–Medicare Part D period.

**Table 1:** Characteristics of the study sample in 2005 (n = 35,102)

	**Pharmacy benefit group**			
	**No coverage (n = 3,939)**	**$150 cap (n = 2,662)**	**$350 cap (n = 19,014)**	**No cap (n = 9,487)**
Female sex, No. (%)	2,183 (55.42)*	1,658 (62.28)*	11,806 (62.09)*	4,984 (52.54)
Age, y, No. (%)				
65-74	1,866 (47.37)*	1,323 (49.70)*	9,983 (52.50)*	5,755 (60.66)
75-84	1,746 (44.33)*	1,080 (40.57)*	7,478 (39.33)*	3,232 (34.07)
≥85	327 (8.30)*	259 (9.73)*	1,553 (8.17)*	500 (5.27)
Race, mean (SE)				
White	0.930 (0.002)*	0.960 (0.001)*	0.919 (0.001)*	0.921 (0.001)
African American	0.052 (0.002)*	0.024 (0.001)*	0.060 (0.001)*	0.057 (0.001)
Status below poverty line, mean (SE)				
<100%	0.106 (0.001)*	0.111 (0.001)*	0.101 (0.000)*	0.099 (0.001)
100-200%	0.183 (0.001)*	0.209 (0.001)*	0.172 (0.000)*	0.169 (0.001)
>200%	0.712 (0.002)*	0.680 (0.002)*	0.727 (0.001)*	0.732 (0.001)
Urban residence, mean (SE)	0.737 (0.005)*	0.577 (0.007)*	0.791 (0.002)	0.797 (0.003)
Income, US dollars, median (SE)	37,573.77 (159.01)*	34,839.85 (111.25)*	38,957.08 (78.64)*	39,501.05 (110.80)
Comorbidity, No. (%)				
Congestive heart failure	507 (12.87)	381 (14.31)	2,656 (13.97)*	1,240 (13.07)
Depression	259 (6.58)*	215 (8.08)	1,465 (7.71)	725 (7.64)
Diabetes	905 (22.98)*	679 (25.51)	4,755 (25.01)	2,464 (25.97)
Hypertension	2,554 (64.84)*	1,892 (71.07)	1,3640 (71.74)*	6,649 (70.09)
Ischemic heart disease	1,110 (28.18)	757 (28.44)	5,551 (29.20)	2,796 (29.47)
Prospective risk score, mean (SE)				
2004	0.825 (0.011)	0.855 (0.014)	0.859 (0.005)	0.844 (0.008)
2005	0.919 (0.012)	0.950 (0.016)	0.942 (0.006)	0.924 (0.009)
2006	1.034 (0.015)	1.040 (0.017)	1.044 (0.007)	1.029 (0.010)
2007	1.153 (0.017)	1.186 (0.020)*	1.176 (0.008)*	1.141 (0.011)
Use of medical services in 2005, mean (SE)				
Outpatient visits, No.	23.146 (0.407)*	25.158 (0.585)	25.134 (0.193)*	25.876 (0.287)
Outpatient costs, US dollars	3,498.249 (92.900)*	3,532.557 (124.010)*	3,741.035 (47.238)	3,869.198 (65.966)
Medical costs, US dollars	6,000.270 (186.796)	5,837.965 (226.731)	6,208.844 (87.559)	6,266.855 (130.414)
Prescriptions of antidementia drugs, mean (SE)				
2004-2007				
Aricept®	0.675 (0.064)*	0.841 (0.090)	0.878 (0.036)	0.908 (0.054)
Razadyne®	0	0	0.008 (0.002)	0.019 (0.005)
Exelon®	0.079 (0.023)*	0.085 (0.032)*	0.100 (0.012)*	0.228 (0.031)
Cognex®	0	0	0	0
Namenda®	0.476 (0.055)	0.343 (0.054)*	0.401 (0.023)*	0.498 (0.038)
Pre–Part D				
Aricept®	0.172 (0.026)*	0.262 (0.039)	0.297 (0.016)	0.335 (0.025)
Razadyne®	0	0	0.008 (0.002)	0.019 (0.005)
Exelon®	0.032 (0.013)*	0.044 (0.018)*	0.040 (0.006)*	0.106 (0.016)
Cognex®	0	0	0	0
Namenda®	0.102 (0.018)	0.084 (0.019)*	0.096 (0.008)*	0.140 (0.015)
Post–Part D				
Aricept®	0.503 (0.046)	0.579 (0.059)	0.581 (0.023)	0.573 (0.033)
Razadyne®	0	0	0	0
Exelon®	0.047 (0.013)*	0.041 (0.016)*	0.060 (0.007)*	0.122 (0.017)
Cognex®	0	0	0	0
Namenda®	0.375 (0.041)	0.259 (0.040)*	0.305 (0.017)	0.357 (0.026)

Abbreviations: SE, standard error.

*differences are statistically significant at p < 0.05 level compared to the No cap group.

**Figure 1:**
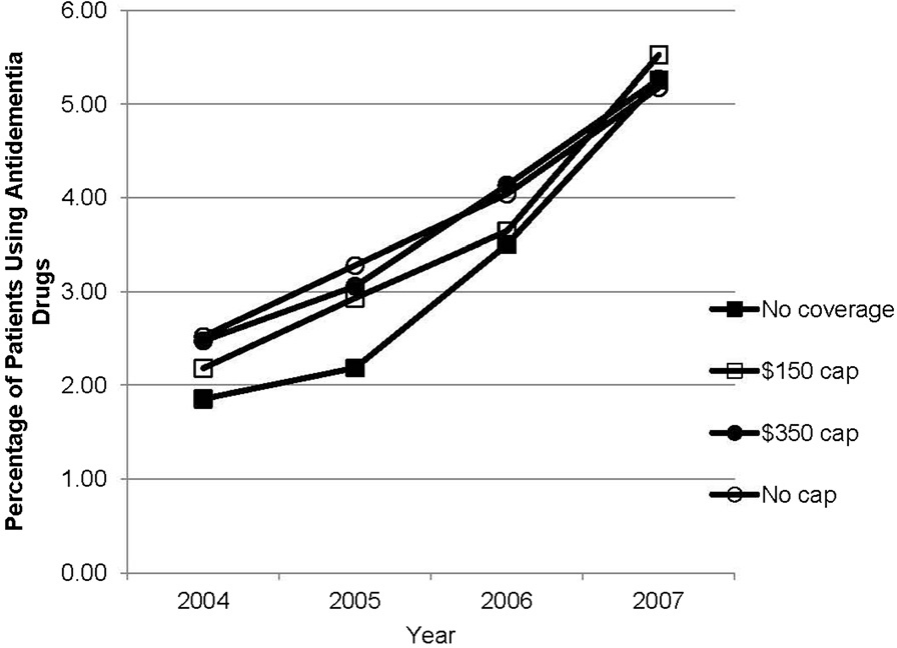
Trends in the proportion of antidementia drug use in the study sample of 35,102 patients, categorized based on drug coverage.

Among the full sample, the proportion of individuals using antidementia drugs increased from 2.4% in 2004 to 5.3% in 2007. During the entire study period, the No coverage group experienced the largest growth in use, with proportions increasing from 1.9% in 2004 to 2.2% in 2005, and to 3.5% in 2006, and 5.3% in 2007. Rates of use post–Part D (2006 and 2007) were significantly different in 2004 (*P* ≤ 0.001) but not in 2005 (*P* = 0.06) (Figure 1). Table 2 displays the pre–post comparison of any use within each coverage group as well as the adjusted odds ratios (AOR) comparing increases in use in the No coverage, $150 and $350 cap groups relative to the No cap comparison group. Each coverage group experienced a statistically significant (*P* < 0.001) increase in the likelihood of antidementia medication use. The magnitude of the increase in odds ratio of use in the No coverage group (AOR = 2.19, 95% [CI], 1.85-2.59) was 38.0% greater than in the No cap group (AOR = 1.59, 95% [CI], 1.47-1.72) whose coverage was stable during the period. The groups with limited coverage pre– Part D ($150 and $350 caps) experienced similar increases in any use to the No cap group that were not significantly different.

**Table 2:** Impact of Medicare Part D on antidementia prescriptions filled annually

**Pharmacy benefit group**	**Proportion with any use pre–Part D**	**Proportion with any use post–Part D**	Adjusted odds ratio* of pre–Part D vs. post–Part D drug use		Comparison of the adjusted odds ratio* of the no coverage, $150 cap, and $350 cap groups with that of the no cap group	
			**AOR (95% CI)**	***P*Value**	**AOR (95% CI)**	***P*Value**
No coverage group	0.02 (0.14)	0.04 (0.20)	2.19 (1.85-2.59)	<0.0001	1.38 (1.14-1.66)	0.0008
$150 cap group	0.03 (0.16)	0.05 (0.21)	1.80 (1.52-2.13)	<0.0001	1.13 (0.94-1.37)	0.1939
$350 cap group	0.03 (0.16)	0.05 (0.21)	1.71 (1.61-1.82)	<0.0001	1.07 (0.97-1.19)	0.1626
No cap group	0.03 (0.17)	0.05 (0.21)	1.59 (1.47-1.72)	<0.0001	[Reference]	

Abbreviations: AOR, adjusted odds ratio CI, confidence interval; SD, standard deviation.

* Analyses were adjusted for sex; age; census-block group-level data concerning race, status below poverty line, residence; health status data (binary variables for congestive heart failure, depression, diabetes, hypertension, and ischemic heart disease); and prospective risk score.

Among the full sample, 3.4% of beneficiaries (n = 1,197) had at least one 30–day antidementia drug prescription pre–Part D. Conditional on any use before Part D, the mean number of 30–day prescriptions filled for these drugs was 6.82 in the pre–Part D period and 9.95 in the post–Part D period across all coverage groups Among those with use pre–Part D, the mean number of 30–day prescriptions of antidementia drugs increased over time from 5.80 to 10.30 in the No coverage group, 5.91 to 8.99 in the $150 cap group, 6.29 to 9.47 in the $350 cap group, and 8.42 to 11.04 in the No cap group.

The adjusted odds ratio of antidementia prescriptions filled post–Part D vs. pre–Part D were statistically significant in all groups (*P* <0.001). In multivariable models that used the No cap group as the reference group and adjusted for covariates, the No coverage group had a 36% greater increase in prescriptions filled (AOR, 1.36; 95% confidence interval [CI], 1.12-1.65; *P* = 0.002), and the $350 cap group had a 15% greater increase (AOR, 1.15; 95% [CI], 1.05-1.27; *P* = 0.003). The increase for the $150 cap group was not statistically significantly different than that for the No cap group (*P* = 0.118) (Table 3).

**Table 3:** Impact of Medicare Part D on antidementia prescriptions filled annually among beneficiaries with any antidementia drug use pre-part D (n = 1,197)

**Pharmacy benefit group**	**Mean No. (SD) of pre–Part D prescriptions**	**Mean No. (SD) of post–Part D prescriptions**	Adjusted odds ratio* of pre–Part D vs. post–Part D prescriptions		Comparison of the adjusted odds ratio* of the no-coverage, $150-cap, and $350-cap groups with that of the no-cap group	
			**AOR (95% CI)**	***P*Value**	**AOR (95% CI)**	***P*Value**
No coverage group	5.80 (6.26)	10.30 (8.56)	1.76 (1.47-2.10)	<0.0001	1.36 (1.12-1.65)	0.0018
$150 cap group	5.91 (5.85)	8.99 (7.35)	1.51 (1.27-1.80)	<0.0001	1.16 (0.96-1.41)	0.1177
$350 cap group	6.29 (5.99)	9.47 (7.92)	1.49 (1.41-1.58)	<0.0001	1.15 (1.05-1.27)	0.0031
No cap group	8.42 (7.07)	11.04 (8.19)	1.30 (1.20-1.40)	<0.0001	[Reference]	

Abbreviations: AOR, adjusted odds ratio CI, confidence interval; SD, standard deviation.

* Analyses were adjusted for sex; age; census-block group-level data concerning race, status below poverty line, residence; health status data (binary variables for congestive heart failure, depression, diabetes, hypertension, and ischemic heart disease); and prospective risk score.

The adjusted odds ratios of the number of cholinesterase inhibitor prescriptions filled post–Part D vs. pre–Part D were statistically significant in all groups (*P* <0.05). In multivariable models that used the No cap group as the comparison group and adjusted for covariates, the No coverage group had 28% greater increase in number of prescriptions filled (AOR, 1.28; 95% confidence interval [CI], 1.02-1.61; *P* = 0.03), and the $350 cap group had a 15% greater increase (AOR, 1.15; 95% CI, 1.05-1.27; *P* = 0.004), relative to the No cap group. The increase for the $150 cap group was not statistically significantly different than that for the No cap group (*P* = 0.141) (Table 4).

**Table 4:** Impact of Medicare Part D on antidementia prescriptions filled annually among beneficiaries with any antidementia drug use pre-part D (n = 1,197) by drug category

**Pharmacy benefit group**	**Mean No. (SD) of pre–part d prescriptions**	**Mean No. (SD) of post–part d prescriptions**	Adjusted ratio* of pre–part D vs. Post–part d prescriptions		Comparison of the adjusted ratio* of the no-coverage, $150-cap, and $350-cap groups with that of the no-cap group	
			**Ratio (95% CI)**	***P*Value**	**Ratio (95% CI)**	***P*Value**
**Cholinesterase inhibitors**						
No coverage groupⱡ	3.87 (4.53)	5.44 (5.33)	1.40 (1.13-1.73)	0.0022	1.28 (1.02-1.61)	0.0323
$150 cap groupⱡ	4.64 (4.71)	5.90 (5.27)	1.27 (1.05-1.53)	0.0125	1.16 (0.95-1.42)	0.1410
$350 cap group**	4.92 (4.68)	6.24 (5.32)	1.26 (1.18-1.34)	<0.0001	1.15 (1.05-1.27)	0.0040
No cap group**	6.45 (5.27)	7.09 (5.59)	1.09 (1.01-1.18)	0.0280	[Reference]	
**Namenda®**						
No coverage group	1.92 (3.57)	4.86 (5.46)	2.50 (1.98-3.16)	<0.0001	1.26 (0.97-1.66)	0.0884
$150 cap group	1.27 (2.87)	3.09 (4.73)	2.38 (1.74-3.25)	<0.0001	1.20 (0.85-1.69)	0.2959
$350 cap group	1.37 (3.05)	3.24 (4.88)	2.33 (2.06-2.64)	<0.0001	1.18 (098.-1.41)	0.0816
No cap group	1.97 (3.74)	3.95 (5.15)	1.98 (1.73-2.27)	<0.0001	[Reference]	

Abbreviations: AOR, adjusted odds ratio; CI, confidence interval; SD, standard deviation.

* Analyses were adjusted for sex; age; census-block group-level data concerning race, status below poverty line, residence; health status data (binary variables for congestive heart failure, depression, diabetes, hypertension, and ischemic heart disease); and prospective risk score.

α Aricept® andExelon®.

** Aricept®, Razadyne, and Exelon®.

The adjusted odd ratios of number of memantine prescriptions filled post–Part D vs. pre–Part D were statistically significant in all groups (*P* <0.0001). In multivariable models that used the No cap group as the reference group and adjusted for covariates, the No coverage group saw a statistically significant increase post–Part D relative to the No cap group (Table 4).

Results from the sensitivity analysis, that was limited to the sub-sample of beneficiaries with a diagnosis of dementia pre–Part D (n = 3,088), yielded similar results. The adjusted ratios of cholinesterase inhibitor prescriptions filled post–Part D vs. pre–Part D showed statistically significant increase in all coverage groups (*P* <0.0001). In multivariable models that used the No cap group as the reference group and adjusted for covariates, the No coverage group had a 80% greater increase in prescriptions filled than the No cap group (adjusted ratio, 1.80; 95% confidence interval [CI], 1.42-2.27; *P* < 0.0001), the $150 cap group had a 29% greater increase in prescriptions filled (AOR, 1.29; 95% confidence interval [CI], 1.02-1.63; *P* < 0.05), and the $350 cap group had a 22% greater increase (AOR, 1.22; 95% CI, 1.09-1.36; *P* = 0.0004) (Table 5).

**Table 5:** Impact of medicare part d on antidementia prescriptions filled annually among beneficiaries with a dementia diagnosis pre-part D (n = 3,088)

**Group based on coverage of drugs in the insurance plan**	**Mean No. (SD) of pre–Part D prescriptions**	**Mean No. (SD) of post–Part D prescriptions**	Adjusted odds ratio* of pre–Part D vs. post–Part D prescriptions		Comparison of the adjusted odds ratio* of the no-coverage, $150-cap, and $350-cap groups with that of the no-cap group	
			**AOR (95% CI)**	***P*Value**	**AOR (95% CI)**	***P*Value**
No-coverage group	1.61 (4.26)	3.88 (7.04)	2.47 (1.99-3.07)	<0.0001	1.80 (1.42-2.27)	<0.0001
$150-cap group	1.75 (4.20)	3.08 (5.99)	1.77 (1.42-2.20)	<0.0001	1.29 (1.02-1.63)	0.0341
$350-cap group	2.34 (4.79)	3.94 (6.86)	1.67 (1.57-1.79)	<0.0001	1.22 (1.09-1.36)	0.0004
No-cap group	3.30 (6.15)	4.66 (7.56)	1.37 (1.26-1.50)	<0.0001	[Reference]	

Abbreviations: AOR, adjusted odds ratio; CI, confidence interval; SD, standard deviation.

* Analyses were adjusted for sex; age; census-block group-level data concerning race, status below poverty line, residence; health status data (binary variables for congestive heart failure, depression, diabetes, hypertension, and ischemic heart disease); and prospective risk score.

### Discussion and conclusions

The growing prevalence of dementia and the significant emotional and economic toll of the condition on patients and families will likely increase the demand for, and spending on, antidementia medications. Our study found substantial increases in the proportion of older Medicare beneficiaries who used these drugs during the study period (2004–2007) and in the number of 30-day prescriptions for these drugs among existing users. Given that the increase in use was greatest among those experiencing the most significant change in drug coverage after Part D’s implementation; the demand for antidementia medications appears to be responsive to changes in out-of-pocket cost. The devastating effects of dementia on patients and families may promote a demand for these medications both in patients who do and do not stand to benefit, particularly when insurance coverage results in low out-of-pocket costs.

Previous studies have shown that Part D has had an impact on the use of drugs with a strong evidence base and clear guidelines recommending their use [21,23,24,27] as well as for drugs without a strong evidence base [21]. We find that the magnitude of the increase in use of antidementia medications associated with Part D is similar to that found for lipid-lowering and antidiabetic medications [27], as well as for drugs to treat heart failure [22] and depression [23].

Medicare Part D plans have several tools at their disposal to improve the appropriateness of prescription drug use among older adults including tiered payment structures for drugs with reduced cost sharing for generic drugs and higher co-payments for brand names drugs, or for drugs with little evidence of effectiveness in some populations. In 2008, donepezil (Aricept®) was available only as a brand name drug and was the seventh most commonly dispensed brand-name drug and the ninth most expensive drug used by Medicare beneficiaries [28]. More than one-third of national Medicare Part D plans had donepezil (Aricept®) on their formularies with no utilization management restrictions, yet, 7 of 47 plans required prior authorization before coverage, while 26 plans had limits on quantity [28]. Little is known about the effects of these utilization management tools on overall use, or on targeting use to those most likely to benefit (e.g., those in early stages of the disease).

Our study had several limitations that deserve mention. First, our study sample consisted of community-dwelling older adults who were living in western Pennsylvania who were enrolled with a single insurer and thus may not be representative of Medicare beneficiaries nationally. Second, it is possible that we did not observe in the claims data all prescriptions filled for those with limited or no coverage before Part D, however, censoring was likely mitigated by limiting the sample to claims data of individuals who filled at least 1 prescription in the insurer’s network of pharmacies. Third, prescription fills may overestimate actual medical use but we do not expect this to vary by coverage group or over time in a way that would bias our estimate. Fourth, we chose to select the sample for our primary analysis based on use of antidementia drugs pre–Part D rather than on the presence of a diagnosis code for dementia, as in the sensitivity analysis. This choice was made based on results from previous work about the inconsistency and underreporting of dementia in claims data [29,30] and due to the fact that use of these drugs for anything other than memory or cognitive complaints in older adults is minimal. Nevertheless it is possible that beneficiaries who use one of these five drugs may be using it for a condition such as Parkinson’s disease. In addition, we were unable to measure or control for the severity of dementia using insurance claims data so we are limited in our ability to comment on appropriateness of use. Our findings of increased used over time, especially for memantine (Namenda®), may be a result of the sample’s worsening cognition and progression to a more severe stage of dementia rather than the impact of enhanced drug coverage through Medicare Part D.

Medicare will need to rely on multiple strategies to achieve its goal of increased access to evidence-based care at the lowest cost. Studies have shown that the introduction of Medicare Part D has had an impact on the use of drugs with both strong and weak evidence for effectiveness and that there are differences in how sensitive demand is to changes in out-of-pocket cost across drug classes. Our findings show a significant increase in the use of antidementia drugs over time among all beneficiaries with steeper increases among those with improved coverage under Medicare Part D.

## Abbreviations

GEE: Generalized estimating equation; HCPCS: Healthcare common procedure coding system; MANOVA: Multivariate analysis of variance

## Competing interests

None of the authors have competing interests to report.

## Authors’ contributions

All authors read and approved the final version of the manuscript. NF developed the study hypothesis, conducted the statistical analysis, and drafted the manuscript. YC conducted the statistical analysis and drafted the manuscript. CT drafted the manuscript. AM assisted with statistical analyses and drafting of the manuscript. ER drafted the manuscript. JD is the senior author and in this role developed the study hypothesis, obtained the dataset, assisted with statistical analysis, and drafting of the manuscript. All authors read and approved the final manuscript.

## Pre-publication history

The pre-publication history for this paper can be accessed here:

http://www.biomedcentral.com/1471-2318/13/37/prepub
